# Association Between Self-Reported Opioid Use and Behavioral/Social Health Characteristics—Arizona, 2020

**DOI:** 10.1007/s11469-024-01276-2

**Published:** 2024-03-18

**Authors:** Samantha Davidson, Mercedeh Javadi, M. Shayne Gallaway

**Affiliations:** 1 Arizona Department of Health Services, Phoenix, AZ USA; 2 Applied Epidemiology Fellowship, Council of State and Territorial Epidemiologists, Phoenix, AZ USA

**Keywords:** Opioids, risk-taking behaviors, Adverse childhood experiences, Population survey

## Abstract

**Background:**

Arizona observed a sharp increase in opioid overdose deaths between 2017 and 2021. Our objective was to better understand the relationship between behavioral/ social characteristics and self-reported opioid misuse among Arizona adults.

**Methods:**

A cross-sectional study design was done using data from the Arizona 2020 Behavioral Risk Factor Surveillance System (BRFSS) (*N* = 10,291). Confidence intervals and p-values were found using chi-square for respondents with and without a self-reported opioid misuse. Logistic regression models were developed for the association between adverse childhood experiences (ACEs), mental health, and risk-taking behaviors (RTBs) and opioid misuse.

**Results:**

Respondents who reported 2–3 ACEs (OR_adjusted_: 4.7; 95% CI: [2.8, 7.9]) and who reported 4 or more ACEs (OR_adjusted_: 8.3; 95% CI: [5.0, 13.6]); respondents who reported poor mental health (OR_adjusted_: 3.3; 95% CI: [2.1, 5.2]); and respondents who reported two or more RTBs (OR_adjusted_: 3.9; 95% CI: [2.5, 6.1]) had higher odds of self-reported opioid misuse compared to those without self-reported opioid misuse.

**Discussion:**

Opioid misuse was found to be associated with poor mental and physical health, increased RTBs, and history of at least two ACEs among Arizona adults in this study. These findings stress the importance of opportunities for targeted prevention in both Arizona adults and youth, including screening for ACEs and RTBs, in early stages of life.

**Supplementary Information:**

The online version contains supplementary material available at 10.1007/s11469-024-01276-2.

## Introduction

The number of U.S. overdose deaths exceeded 100,000 for the first time by July 2021 (Centers for Disease Control and Prevention, 2022a). In 2020, over 70% of the overdose deaths in the United States involved an opioid (Centers for Disease Control and Prevention, 2021). From 2019 to 2020, the United States observed an increase of opioid-involved death rates by 38% (Centers for Disease Control and Prevention, 2022b). Opioid deaths continue to increase as the use of synthetic opioids (e.g., fentanyl) becomes more widespread. Arizona has observed similar trends in recent years (2017–2021) (Arizona Department of Health Services, 2023b). In 2017, the Arizona Governor declared a statewide emergency in order to reduce opioid deaths (Arizona Department of Health Services, 2017). Arizona has taken substantial actions to address the epidemic that included establishing the Opioid Assistance and Referral (OAR) Line, a hotline that offers opioid information, resources, and referrals for providers and the public (Arizona Department of Health Services, 2023a), establishing the overdose fatality review committee (Arizona Department of Health Services, 2022), and launching an online interactive dashboard (Arizona Department of Health Services, 2023b). The overall trend of opioid overdose deaths increased significantly from 2017 to 2020, and while it was stable from 2020 to 2021, there were over 2,000 opioid deaths during 2021 in Arizona, a two-fold increase compared with 2017 (Arizona Department of Health Services, 2023b). Opioid use disorder and fatal opioid overdoses have tremendous economic costs. In 2017, the cost of opioid use disorder and fatal opioid overdose in the U.S. was estimated at over $1 trillion, of which $21.8 billion was in Arizona (Luo et al., 2021).

Preventing opioid misuse and overdose is a multifaceted issue. Many factors can contribute to an individual’s risk of misuse and overdose, including comorbid mood and anxiety disorders (Grant et al., 2004; Quello et al., 2005). Treating comorbid mood or anxiety disorders may reduce cravings and improve treatment for substance use disorder (Grant et al., 2004). Interventions to treat opioid use disorder may include medication-assisted treatment (MAT) with opioid agonists including methadone and buprenorphine, or non-opioid agents including α2-adrenergic agonists (Schuckit, 2016; Stotts et al., 2009; Substance Abuse and Mental Health Services, 2005) coupled with non-pharmacologic treatments such a behavioral therapy to improve medication compliance and target problems not addressed with medication alone (Sofuoglu et al., 2018). Treating comorbid behavioral health and substance use disorders is important because they have the potential to reduce MAT effectiveness (Sofuoglu et al., 2018).

There are several known risk factors for opioid misuse and overdose including past substance use, psychiatric comorbidities, and certain social and family environments (Webster, 2017). Psychiatric comorbidities include major depression, dysthymia, generalized anxiety disorder, panic disorder, and post-traumatic stress disorder (PTSD) (Amari et al., 2011; Davis et al., 2017; Seal et al., 2012; Sullivan et al., 2006). Individuals with adverse childhood experiences (ACEs) may also be at increased risk of poor mental health and opioid misuse (Guarino et al., 2021; Matjasko et al., 2022). ACEs can include any potentially traumatizing events occurring in childhood, such as physical or sexual abuse, neglect, or having an incarcerated parent. Much research has gone into the long-lasting effects of such events on the health of children who experience them. Importantly, there has also been longitudinal research, looking at how adults who experienced ACEs as children have fared as they have grown up. For example, Mersky, Topitzes, and Reynolds examined data from the Chicago Longitudinal Study, which looks at individuals born in 1979 or 1980 (Mersky et al., 2013). They found that individuals who had experienced multiple ACEs were more likely to report 3 or more and 4 or more poor health, substance use, and mental health outcomes (Mersky et al., 2013). Several studies on risk taking behavior suggest opioid misuse may be related to risky behavior (e.g., having unprotected sex, smoking, reckless driving), but research is lacking among some adult populations (Bhatia et al., 2020; Sanchez-Roige et al., 2021). A cohort study of 132,113 participants examined the genome-wide association of problematic opioid use, and found it is positively genetically correlated with risk-taking, but that it does not simply reflect a genetic tendency towards risky behavior (Sanchez-Roige et al., 2021). An analysis of high school students found that adolescents who misused opioids were more likely to have engaged in risky behaviors compared with those who did not misuse opioids (Bhatia et al., 2020). This research identified a need for more screening measures for opioid misuse in adolescents so early interventions can be implemented and further research to investigate the relationship between risky-behavior, opioid misuse, and other adverse health outcomes.

There are several gaps in and areas identified in the current literature for future research. While the relationship between ACEs and opioid misuse has been extensively studied, more work is required to ensure providers perform ACEs screening and perform opioid misuse risk assessments on all patients (Deol et al., 2023). The current study aims to support those conclusions. The relationship between mental health conditions and opioid misuse is also well studied, but the literature has also identified needs for the treatment of comorbid conditions as a preventative measure for treating opioid misuse disorder (Deol et al., 2023), which this study also aims to address. The literature is lacking research on the relationship of risk-taking behaviors and opioid misuse, especially risk-taking behaviors in adulthood. One study in an adult population examined risk behaviors among persons civilly committed for opioid use, however this research aimed to determine if an individual’s opioid use poses a risk for serious harm (Christopher et al., 2022). Research on this topic has mainly been limited to adolescent populations (Clayton et al., 2019; Bhatia et al., 2020) or in studies where opioid misuse is the exposure (D’Amico et al., 2021). This study aims to examine the associations between risk-taking behaviors and opioid misuse among adults.

Our objective was to better understand the relationship between ACEs, mental health issues, and risk-taking behavior with self-reported opioid misuse among Arizona adults.

## Methods

A cross-sectional data analysis was completed using the Arizona 2020 Behavioral Risk Factor Surveillance System (BRFSS) (Arizona Department of Health Services, 2020), a representative, state-based telephone survey that recruits residents via landline or cellular telephone (*N* = 10,291). The BRFSS was created by the Centers for Disease Control and Prevention and has been used to collect data about United States residents since 1984, with categories of interest including health conditions, risk behaviors, and preventive services (Centers for Disease Control and Prevention, 2014). The BRFSS also collects health-related quality of life (HRQOL) data through “Healthy Days” measures that the CDC both developed and validated (Moriarty et al., 2003). The combined response rate for landline and cell phone samples was 46.8%.

### Opioid Misuse

A comparison of demographic, healthcare, and personal characteristics was completed among persons categorized with and without self-reported opioid misuse. Three questions asking about opioid misuse added to the Arizona BRFSS were used as an indicator for opioid misuse (i.e., a yes response to any 1 of 3 questions indicated opioid misuse). The three questions were as follows: (1) “In the past year, did you use a prescription pain medication that was not prescribed specifically for you by a doctor, dentist, nurse practitioner, or healthcare providers?” (2) “In the past 12 months, did you shoot up or inject any drugs other than those prescribed for you?” and (3) “In the past year have you felt dependent on prescription pain medication or experienced trouble getting off of the medication when you no longer needed it for medical reasons?” Respondents with a “yes” to any of the previous questions were considered to have opioid misuse. Those who answered “no,” “don’t know,” or refused to answer were considered to have no opioid misuse.

### Demographic Characteristics

The following sociodemographic characteristics were examined: sex; age group (18–24, 25–34, 35–44, 45–54, 55–64, and 65 years or older); race/ ethnicity (non-Hispanic White, Black, Asian/Pacific Islander, American Indian/Alaska Native, Hispanic, Other/Multiracial); educational attainment (less than high school, high school, and greater than high school); marital status (married/partnership or not married); employment status (employed, unemployed, or not in labor market, including homemaker, student, retired, and unable to work); and annual household income (<$15,000; $15,000 -$24,999; $25,000-$34,999; $35,000-$50,000; or >$50,000).

### Health Characteristics

Healthcare services access was assessed via healthcare coverage (including health insurance, prepaid plans, or government plans), personal doctor or healthcare provider, and whether they reported needing to see a doctor but were unable due to costs. Respondents reported how many days during the past 30 days their physical health was not good, which were categorized as: 0 days, 1–13 days, and 14 + days when physical health was not good.

### Adverse Childhood Experiences (ACEs), poor Mental Health, and risk-taking Behaviors (RTBs)

Respondents self-reported ACEs they had during their childhood, including being sworn at, being touched/being forced to touch someone else sexually, being forced to have sex, being physically hurt, watching one’s parents physically hurt each other, and whether their parents were divorced/separated. Respondents also reported details about persons they lived with in their childhood, including whether they lived with “anyone depressed, mentally ill, or suicidal; a problem drinker or alcoholic,” “anyone who used illegal drugs or prescriptions,” or “anyone who served time in prison or jail.” The total number of self-reported ACEs were categorized as follows: 0, 1, 2–3, or 4 + ACEs.

Two questions were asked to determine mental health status. Respondents reported the number of days (in the past month) their mental health was not good, which were categorized as 0, 1–13, or 14 or more days their mental health was not good. Secondly, respondents reported whether they had ever been told by a healthcare provider they had a depressive disorder. Respondents were categorized as having poor mental health if they had 14 + days where their mental health was not good in the past 30 days or if they have ever been told by a physician that they had a depressive disorder.

Respondents self-reported five types of RTBs, including binge drinking (“how many times during the past 30 days did you have 5 or more drinks for men or 4 or more drinks for women”), smoking marijuana (“in the past year have you ever used marijuana or hashish”), smoking tobacco (four-level smoking status: every day smoker, someday smoker, former smoker, non-smoker), whether they always wear a seatbelt (always wear seatbelts variable), and whether they drink and drive (reported having driven at least once when perhaps had too much to drink). The total number of RTBs were summarized and categorized as follows: 0, 1, or 2 + RTBs.

### Statistical Analysis

Analyses were done using SAS survey procedures (SAS version 9.4, SAS Institute Inc., Cary, North Carolina, USA) due to the sampling design of BRFSS. Population numbers were estimated using weighting and stratification variables. Confidence intervals and p-values (< 0.05 used for significance) were found using chi-square for respondents with and without opioid misuse. Two logistic regression models were developed for the associations ACEs, mental health, and RTBs separately with opioid misuse. The first model was unadjusted for any confounding variables. The second model adjusted ACEs for possible confounding from sex, race, marital status, education level, employment status, and income; and mental health and RTBs were adjusted for potential confounding from sex, race, marital status, education level, and income. Best-fit models were determined through backward deletion of least significant variable until all variables remaining in the model had a P-value of 0.20 or less, thus adjusting for different variables for ACEs versus mental health and RTBs.

### Ad hoc Analysis

An ad hoc analysis was performed to determine whether there was a difference in magnitude between the three variables used to define opioid misuse and ACEs, poor mental health, and risk-taking behaviors. Unadjusted odds ratios and 95% confidence intervals were used to determine significance.

## Results

Of the 10,291 respondents (weighted *N* = 5,753,714), 180 (weighted *N* = 115,277) reported using a prescription pain medication that was not specifically prescribed for them; 29 (weighted *N* = 23,993) reported injecting- any drugs not prescribed to them; and 51 (weighted *N* = 26,540) reported feeling dependent on prescription pain medication. Overall, 2.6% of weighted respondents self-reported at least one example of opioid misuse (Table 1).

**Table 1:** Comparison of demographic characteristics between persons reporting opioid misuse, BRFSS, Arizona, 2020

Characteristic	Self-Reported Opioid Misuse				No Self-Reported Opioid Misuse				p-value
	N	Weighted N	%	95% CI	N	Weighted N	%	95% CI	
**Total**	244	149,497	2.6		10,047	5,604,217	97.4		
**Sex**									**0.003**
Male	129	92,255	61.7	53.5–69.9	4,634	2,743,810	49.0	47.5–50.4	
Female	115	57,242	38.3	30.1–46.5	5,413	2,860,407	51.0	49.6–52.5	
**Age group (years)**									**0.053**
18–24	15	18,768	12.6	5.7–19.4	616	690,593	12.3	11.2–13.4	
25–34	42	39,143	26.2	18.1–34.3	1,047	969,887	17.3	16.2–18.4	
35–44	35	28,590	19.1	11.7–26.6	1,204	895,485	16.0	14.9–17.1	
45–54	42	21,576	14.4	9.1–19.8	1,531	846,359	15.1	14.2–16.0	
55–64	41	16,095	10.8	6.6–14.9	1,896	861,196	15.4	14.4–16.3	
65+	69	25,326	16.9	11.3–22.6	3,753	1,340,696	23.9	22.8–25.0	
**Race/ Ethnicity**									**< 0.001**
White, Non-Hispanic	137	71,076	47.5	38.8–56.3	6,802	3,260,160	58.2	56.7–59.6	
Black, Non-Hispanic	5	5,260	3.5	0.0–7.0	263	247,906	4.4	3.8–5.1	
Asian/Pacific Islander	1	812	0.54	0.0-1.6	160	206,790	3.7	3.0-4.4	
American Indian/ Alaska Native	23	14,126	9.4	4.1–14.8	592	195,804	3.5	3.1–3.9	
Hispanic	68	55,571	37.2	28.6–45.7	1,828	1,584,736	28.3	26.9–29.6	
Other/ Multiracial	10	2,652	1.8	0.1–3.4	402	108,820	1.9	1.7–2.2	
**Marital Status**									**0.002**
Married/Partnership	102	61,775	41.3	32.7–50.0	5472	3,064,689	55.3	53.9–56.8	
Not Married	142	87,722	58.7	50.0-67.3	4,451	2,473,265	44.7	43.2–46.1	
**Education Level**									**0.001**
Did Not Graduate High School	31	34,638	23.2	14.9–31.5	758	744,928	14.1	12.2–14.5	
High School Graduate	83	46,993	31.4	28.8–39.1	2,385	1,404,065	25.8	24.0-26.4	
Greater than High School Graduate	130	67,866	45.4	36.7–54.1	8,802	3,424,221	61.4	60.0-62.9	
**Employment Status**									**0.073**
Employed*	98	71,383	48.0	39.2–56.8	4,517	2,986,613	54.8	53.3–56.2	
Out of Work/ Unable to Work	57	27,517	18.5	12.0-24.9	1,227	65,927	12.1	11.2–13.0	
Other **	88	49,926	33.5	25.1–42.0	8,022	1,808,847	33.2	31.8–34.5	
**Income Level**									**0.002**
<$15,000	42	22,929	17.9	11.3–24.5	790	379,738	8.6	7.7–9.5	
$15,000 -$24,999	59	26,067	20.4	13.4–27.3	1,313	740,291	16.8	15.6–18.0	
$25,000-$34,999	24	14,377	11.2	4.7–17.7	809	421,202	9.5	8.6–10.5	
$35,000-$50,000	26	11,558	9.0	4.6–13.5	1,177	657,825	14.9	13.8–16.1	
>$50,000	67	52,967	41.4	31.9–50.9	3,911	2,212,960	50.2	48.6–51.7	

Chi square used to obtain p-value for significance estimation (< 0.05), as well as 95% Confidence Interval (CI)

* Wages, self-employed

**Homemaker, Student, Retired

Compared with respondents who did not report opioid misuse, those reporting opioid misuse were noticeably more likely to report more 14 or more days of poor health compared with persons who did not report opioid misuse (19.3% vs. 9.6%) (Table Table 2).

Respondents who reported opioid misuse were significantly more likely to report having all ACEs compared with those who did not report opioid misuse (< 0.05) (Table Table 2). The total number of ACEs reported was substantially higher among those reporting opioid misuse than those who did not report opioid misuse [0 (16.3% vs. 48.9%), 1 (9.7% vs. 17.6%), 2–3 (29.6% vs. 18.0%) or 4 or more (44.4% vs. 15.4%) ACEs] (Table 2).

**Table 2:** Comparison of health characteristics between persons reporting opioid misuse, BRFSS, Arizona, 2020

Characteristic	Self-Reported Opioid Misuse				No Self-Reported Opioid Misuse				p-value
	N	Weighted N	%	95% CI	N	Weighted N	%	95% CI	
**Total**	244	149,497	2.6		10,047	5,604,217	97.4		
**Healthcare Coverage**									**< 0.001**
Yes	197	110,867	74.6	66.6–82.5	8,948	4,869,554	85.7	84.6–86.8	
No	26	37,818	25.4	17.5–33.4	1,035	831,682	14.3	13.2–15.4	
**Personal Doctor**									**0.045**
Yes	171	92,407	62.8	53.9–71.7	7,709	3,960,842	71.4	70.1–72.7	
No	70	54,760	37.2	28.3–46.1	2,266	1,587,217	28.6	27.3–29.9	
**Forego Doctor Due to Cost**									**0.003**
Yes	48	29,631	19.8	13.1–26.5	971	639,775	11.5	10.5–12.4	
No	196	119,866	80.2	73.5–86.9	9,041	4,947,019	88.5	87.6–89.5	
**Poor Physical Health**									**< 0.001**
0 days	128	88,766	59.4	51.5–68.2	6,779	3,936,069	70.2	70.8–73.3	
1-13- days	53	30,558	20.4	13.9–27.3	1,859	985,085	17.6	17.0-19.1	
14 + days	57	28,917	19.3	13.2–25.8	1,153	538,827	9.6	9.1–10.7	

Chi square used to obtain p-value for significance estimation (< 0.05), as well as 95% Confidence Interval (CI)

Respondents who reported opioid misuse were significantly more likely to report their mental health was not good for 14 or more days in the past month, compared with those who did not report opioid misuse (28.9% vs. 13.1%) (Table 3). Respondents who reported opioid misuse were also considerably more likely to report a lifetime depressive disorder diagnosis (34.5%), compared with those who did not report opioid misuse (16.9%) (Table 3). Overall, 21.6% of respondents who reported opioid misuse were classified as having poor mental health and 7.4% of respondents who did not report opioid misuse were classified as having poor mental health (Table 3).

**Table 3:** Comparison of adverse childhood experiences (ACEs), poor mental health and risk-taking behaviors (RTBs) between persons reporting opioid misuse, BRFSS, Arizona, 2020

	Self-Reported Opioid Misuse				No Self-Reported Opioid Misuse				p-value
	N	Weighted N	%	95% CI	N	Weighted N	%	95% CI	
Adverse Childhood Experiences (ACEs)									
Lived with anyone depressed, mentally ill, or suicidal	81	61,348	41.3	(32.4, 50.2)	1,261	720,079	17.0	(15.7, 18.1)	< 0.001
Lived with a problem drinker/alcoholic	102	70,476	47.3	(38.4, 56.1)	1,878	1,008,961	23.6	(22.2, 25.0)	< 0.001
Lived with anyone who used illegal drugs or abused prescriptions	58	49,335	33.2	(24.3, 42.0)	797	517,288	12.1	(11.0, 13.2)	< 0.001
Lived with anyone who served time in prison or jail	53	43,329	29.1	(20.5, 37.6)	596	410,460	9.6	(8.6, 10.6)	< 0.001
Parents divorced/separated	95	74,306	50.2	(41.4, 59.0)	2,198	1,359,360	32.0	(30.5, 33.5)	< 0.001
Parents beat each other up	65	49,382	33.1	(24.6, 41.7)	1,247	704,063	16.9	(15.6, 18.1)	< 0.001
Parent physically hurt you in any way	98	62,645	42.3	(33.5, 51.1)	2,036	1,102,191	26.3	(24.8, 27.7)	< 0.001
Parent swear at you	128	79,509	53.7	(44.9, 62.5)	2,704	1,483,307	35.5	(33.9, 37.1)	< 0.001
Sexual/other touching	60	36,156	24.4	(16.8, 32.1)	924	467,401	11.2	(10.2, 12.2)	< 0.001
Anyone make you touch them	45	22,737	15.6	(9.2, 22.0)	645	323,998	7.7	(6.9, 8.6)	0.002
Anyone ever force you to have sex	28	14,187	9.6	(4.4, 14.7)	385	198,601	4.7	(4.1, 5.4)	0.013
**Number of ACEs**									< 0.001
0	43	24,380	16.3	(10.2, 22.4)	4,883	2,742,205	48.9	(47.5, 50.3)	
1	34	14,534	9.7	(5.1, 14.3)	1,884	988,214	17.6	(16.6, 18.7)	
2–3	74	44,222	29.6	(21.7, 37.4)	1,758	1,007,954	18.0	(16.9, 19.1)	
4+	93	66,362	44.4	(35.6, 53.2)	1,522	865,843	15.4	(14.4, 16.5)	
**Mental health Status**									
14 + days (in past month) mental health not good	61	41,284	28.9	(20.6, 37.3)	1,240	717,419	13.1	(12.2, 14.0)	< 0.001
Lifetime self-reported depressive disorder diagnosis	84	50,777	34.5	(26.1, 42.9)	1,783	941,653	16.9	(15.9, 17.9)	< 0.001
**Mental health** ^**a**^									< 0.001
Poor	49	32,351	21.6	(14.2, 29.1)	711	416,302	7.4	(6.7, 8.2)	
**Risk-Taking Behaviors (RTBs)**									
Binge drinking (past 30 days)	52	39,932	53.9	(41.5, 66.2)	1,050	699,353	28.0	(26.1, 30.0)	< 0.001
Marijuana or hashish used (past year)	82	57,698	43.2	(34.1, 52.4)	1,096	681,672	17.0	(15.7, 18.2)	< 0.001
Current or former smoker	147	83,718	56.5	(47.7, 65.3)	3,980	2,007,191	38.6	(37.2, 40.0)	< 0.001
Doesn’t always wear a seatbelt	49	36,965	24.7	(16.6, 32.8)	1,139	585,343	11.5	(10.5, 12.5)	< 0.001
History of drinking and driving	2	4,556	5.7	(0.0, 13.3)	75	44,757	1.7	(1.2, 2.2)	0.077
**Number of RTBs** ^**b**^									< 0.001
0	61	41,516	27.8	(19.9, 35.6)	4,800	2,803,715	50.0	(48.6, 51.4)	
1	84	40,509	27.1	(19.5, 34.7)	3,589	1,847,456	33.0	(31.7, 34.3)	
2+	99	67,472	45.1	(36.4, 53.9)	1,658	953,046	17.0	(15.9, 18.1)	

Abbreviations: OR- Odds Ratio, CI- Confidence Interval, ACE- Adverse Childhood Event, RTB- Risk-Taking Behavior

Definitions: ^a^Poor Mental Health- Reported 14 + days mental health was not good in the last 30 days or Reported ever being told you have a depressive disorder (including depression, major depression, dysthymia, or minor depression); ^b^Risk-Taking Behavior- Current or former smoker, does not always wear a seatbelt, history of drinking and driving, binge drinking n the past 30 days, or marijuana use in the past year

Compared with respondents who did not report opioid misuse, those who reported opioid misuse were substantially more likely to report binge drinking in the past 30 days (53.9% vs. 28.0%), using marijuana or hashish in the past year (43.1% vs. 17.0%), being a current or former smoker (56.5% vs. 38.6%), not always wear a seatbelt (24.7% vs. 11.5%), and/or a history of drinking and driving (5.7% vs. 1.7%) (Table 3). The total number of reported RTBs was greater among persons reporting opioid misuse compared with persons not reporting opioid misuse [0 (27.8% vs. 50.0%), 1 (27.1% vs. 33.0%), 2 or more (45.1% vs. 17.0%) RTBs] (Table 3).

Respondents who reported 2–3 ACEs had an almost 5 times higher odds of self-reported opioid misuse compared with those who reported zero ACEs (aOR: 4.7; 95% CI: [2.8, 7.9]), and those who reported 4 or more ACEs had over 8 times higher odds of opioid misuse (aOR: 8.3; 95% CI: [5.0, 13.6]) (Table 4). Respondents who reported poor mental health had an over 3 times higher odds of opioid misuse compared with those who did not report poor mental health (aOR: 3.3; 95% CI: [2.1, 5.2]) (Table 4). Respondents who reported two or more RTBs had an almost 4 times higher odds of opioid misuse compared with those who reported no RTBs (aOR: 3.9; 95% CI: [2.5, 6.1]) (Table 4).

**Table 4:** Adjusted and Unadjusted Odds Ratios (OR) and 95% Confidence Intervals (95% CI) of Self-Reported Opioid Misuse, BRFSS, Arizona, 2020

	Unadjusted^1^		Adjusted^2^	
	OR	95% CI	aOR	95% CI
**Number of ACEs**				
0	1	Ref.	1	Ref.
1	1.7	(0.9, 3.1)	1.6	(0.8, 3.0)
2–3	4.9	(2.9, 8.3)	4.7	(2.8, 7.9)
4+	8.6	(5.2, 14.2)	8.3	(5.0, 13.6)
**Mental health** ^**a**^				
Not poor	1	Ref.	1	Ref.
Poor	3.4	(2.2, 5.4)	3.3	(2.1, 5.2)
**Number of Risk-Taking Behaviors** ^**b**^				
0	1	Ref.	1	Ref.
1	1.5	(0.9, 2.4)	1.4	(0.9, 2.2)
2+	4.8	(3.1, 7.4)	3.9	(2.5, 6.1)

Abbreviations: OR- Odds Ratio, CI- Confidence Interval, ACE- Adverse Childhood Event, RTB- Risk-Taking Behavior

^1^Logistic regression of ACEs, Mental Health, or Risk-Taking Behaviors and opioid misuse

^2^ ACEs- additionally adjusted for sex, race, marital status, education level, employment status, and income; Mental Health- additionally adjusted for sex, race, marital status, education level, and income; Number of RTBs- additionally adjusted for sex, race, marital status, education level, and income

Definitions: ^a^Poor Mental Health- Reported 14 + days mental health was not good in the last 30 days or Reported ever being told you had a depressive disorder (including depression, major depression, dysthymia, or minor depression); ^b^Risk-Taking Behavior- Current or former smoker, does not always wear a seatbelt, history of drinking and driving, binge drinking in the past 30 days, or marijuana use in the past year

Respondents who reported 2–3 ACEs, 4 + ACEs, poor mental health, and 2 + RTBs had higher odds of misuse of prescription pain medication than those who reported no misuse history (Supplemental Table 1). Respondents who reported 2–3 ACEs, 4 + ACEs, poor mental health, 1 RTB, and 2 + RTBs had higher odds of history of injection of non-prescription drugs. Respondents who reported 4 + ACEs and poor mental health had higher odds of feeling dependent on prescription pain medication (Supplemental Table 1).

## Discussion

The current study investigated the complex relationship between behavioral/social factors and opioid misuse to increase understanding of how these are related and the role of each among Arizona adults. This study identified multiple factors significantly associated with self-reported opioid misuse, including poor physical and mental health, increased number of risk-taking behaviors, and multiple ACEs. Although these correlates are consistent with those observed in studies examining the relationship between mental health (Cruden & Karmali, 2021; Mackesy-Amiti et al., 2015), ACEs (Merrick et al., 2020; Swedo et al., 2020), and risk-taking behaviors (Bhatia et al., 2020; Clayton et al., 2019) with opioid misuse, we observed a consistently high likelihood of all these in the same population reporting opioid misuse compared to adults who did not report opioid misuse. This population with multiple negative behavioral and social health outcomes was also more likely to report challenges associated with accessing medical services.

Our study is consistent with previous studies which have found an increased risk of opioid misuse among persons with ACEs (Merrick et al., 2020; Swedo et al., 2020). ACEs are associated with younger age of opioid initiation, injection drug use, and the likelihood of experiencing overdose (Quinn et al., 2016; Stein et al., 2017). The results in the current analysis are similar to previous studies which identified that the odds of opioid misuse as an adult increased with four or more different experiences of trauma before age 18 (Quinn et al., 2016). In general, increased ACEs have been associated with increases in the probability of all substance use, including alcohol and tobacco (Schwartz et al., 2023), as well as risk-taking behaviors such as unprotected sex (VanderEnde et al., 2018). ACEs are also correlated with poor mental and physical health (Brennenstuhl & Fuller-Thomson, 2015; Cambron et al., 2014; Monnat & Chandler, 2015).

Previous studies have examined risk-taking behavior and opioid misuse in adolescents and young adults in various settings (Bhatia et al., 2020; Clayton et al., 2019). Early initiation of opioid misuse is a significant risk factor for the development of substance use disorder, and persons engaging in other risk-taking behaviors have been shown to initiate drug use at an earlier age (Levy, 2019; Rosenbaum & Kandel, 1990; Trenz et al., 2012). Our study was able to confirm that engagement in an increasing number of risk-taking behaviors, including polysubstance use (tobacco, marijuana, and binge drinking) was associated with increased odds of opioid misuse. Risk-taking behavior in adolescents has been studied thoroughly as an outcome, with many reviews looking at mental health, coping styles, social stress, etc. as risk factors for risk-taking behaviors (Khodarahimi & Fathi, 2016; Reynolds et al., 2013), thus demonstrating a potential upstream intervention for such behavior and correlates such as opioid misuse. This may be why campaigns targeting adolescent risk-taking behaviors target aspects such as social stress and mental health as well, such as in the “Truth” anti-smoking/vaping/opioid campaign (Truth Initiative, 2023). In a page dedicated to the mental health effects of vaping, a list of youth-targeted resources is provided that include meditation apps, an MTV-backed mental health website, and text-based peer support (Truth Initiative, 2023). The Truth campaign also targets misleading marketing and offers young adults opportunities to elicit change, for themselves and for their community, thus making smoking/vaping/opioids something that not only harm oneself, but the world as well (Truth Initiative, 2023).

Poor health-related quality of life is greatly correlated with opioid misuse, as evidenced by the significant association observed in this study between physical and mental health and opioid misuse. Previous studies have observed a significant increased comorbidity of substance use disorders and chronic diseases (Wu et al., 2018). This is particularly problematic because chronic diseases can impact the successful likelihood of accessing, utilizing, and improving overall health (Ducat et al., 2014; McLellan & Woodworth, 2014; Wu et al., 2018). Characterizing patterns of comorbidity may help inform case management and treatment efforts for persons misusing opioids or who have a substance use disorder, and recognizing commonly correlated comorbid conditions may aid in identifying those at risk for future opioid misuse. Respondents in our study who reported opioid misuse were less likely to report healthcare coverage and more likely to forego medical care due to cost, which are both factors that can lead to poor mental and physical health.

### Limitations

This study was not without limitations. First, recall bias may be present in this study. Among those who self-reported opioid misuse, 22% reported poor mental health and 84% reported at least one ACE. Individuals with depression or chronic stress may be more likely to overreport ACEs than those who did not develop depression or chronic stress (Colman et al., 2016), therefore the number of ACEs reported may be overestimated among those who reported poor mental health. Additionally, individuals with more “neurotic” personalities are more likely to overreport ACEs than individuals with “agreeable” personalities when comparing prospective and retrospective accounts of ACEs (Reuben et al., 2016). Second, some individuals may be less likely to self-report illicit use of opioids because of a desire to give socially desirable responses. In addition, individuals may be less likely to report opioid use in a research setting (Khalili et al., 2021). Individuals may also desire to give socially desirable responses to questions regarding ACEs, especially since BRFSS surveys are conducted via phone and the respondents may not be alone when responding to the survey. Third, the Arizona State-Added Section on Opioids is not included in the Arizona BRFSS every year or included by all states. For this reason, we did not make temporal or inter-state comparisons with self-reported opioid misuse in this analysis. Fourth, selection bias may be present, as the BRFSS excludes incarcerated individuals. Incarcerated individuals may face higher rates of substance use than non-incarcerated individuals (National Institute on Drug Abuse, 2020). Fifth, nonresponse bias may be present in this study. The response rate is the number of respondents who completed the survey as a proportion of all eligible and likely-eligible people. The median survey response rate for all states, territories and Washington, DC, in 2020 was 47.9 and ranged from 34.5 to 67.2. The response rate for Arizona had a median of 46.8 (Centers for Disease Control and Prevention, 2021). Sixth, we defined opioid misuse as anyone who reported in the past year that they used prescription pain medication that was not prescribed for them, injected any drugs other than those prescribed for them, and/ or felt dependent on prescription pain medication or experienced trouble getting off of the medication when you no longer needed it for medical reasons. The question “In the past 12 months, did you shoot up or inject any drugs other than those prescribed for you?” does not specify injection of opioids. Those who answered “Yes” to this question may be referring to drugs other than opioids, including stimulants. Therefore, the number of respondents who we defined as having self-reported opioid misuse may be an overestimate. The authors determined it best to include this measure as an indicator of opioid misuses as it may capture some cases of opioid misuse not captured by the other two questions. Additionally, respondents who answered “yes” to any of the three opioid misuse questions were considered to have opioid misuse, and those who answered “no,” “don’t know,” or refused to answer were considered to have no opioid misuse. Because we included respondents who responded “don’t know” or refused to answer in the reference group, our associations may be overestimates or underestimates, depending on if those respondents were more or less likely to self-report opioid misuse. In the future, we could perform sensitivity analyses to determine what effect removing these respondents from the reference group would have on results. However, those who responded “don’t know” or refused to answer the opioid questions made up less than 1% of responses for two of the questions, and less than 3% for the third question. Another limitation of this analysis is that we limited our analysis of ACEs to ACE scores instead of ACE theme categories: abuse (emotional abuse, physical abuse, sexual abuse), household challenges (intimate partner violence, substance abuse in the household, mental illness in the household, parental separation or divorce, incarcerated household member), and neglect (emotional neglect, physical neglect) (National Center for Injury Prevention and Control, 2023). Future analyses could include these three categories to account for differential effects from certain ACEs (McLaughlin et al., 2019). Another limitation is our use of backward deletion to develop best-fit models. There is ongoing discussion regarding the appropriateness of stepwise methods for regression (Ruengvirayudh & Brooks, 2016; Smith, 2018). However, due to high collinearity of our variables, a stepwise method was deemed appropriate. Lastly, marijuana, tobacco, and binge drinking were included as RTBs, which may be considered polysubstance use. Polysubstance use is known to be associated with opioid misuse, which may artificially inflate association.

## Conclusions

Opioid misuse was found to be associated with poor mental and physical health, increased risk-taking behaviors, and history of ACEs among Arizona adults in this study. Arizona adults who reported opioid misuse face unique circumstances including increased barriers to care and worsened mental and physical health compared to adults with no history of opioid misuse, and may require resources to address risk taking behaviors and ACEs- tailored specifically to individuals with SUD (Clayton et al., 2019; Levy, 2019). There may be opportunities for targeted opioid prevention for youth experiencing ACEs and those who demonstrate risk-taking behaviors to prevent initial use. Mental and physical health services can prevent opioid misuse (e.g., mental health screening during regular primary care visits, more accessibility to mental health services when indicated) (Cruden & Karmali, 2021; Mackesy-Amiti et al., 2015; Wu et al., 2018). Other states may consider including the State-Added Section on Opioids in their state BRFSS so that they can learn more about associated health and risk-factors of opioid use, and comparisons can be made between states.

## Electronic supplementary material

Below is the link to the electronic supplementary material.

Supplementary Material 1

## Data Availability

The data used in this analysis is the Arizona, 2020 subset of data from the Behavioral Risk Factor Surveillance System (BRFSS).

## References

[CR1] Amari, E., Rehm, J., Goldner, E., & Fischer, B. (2011). Nonmedical prescription opioid use and mental health and pain comorbidities: A narrative review. *Canadian Journal of Psychiatry*, *56*(8), 495–502. 10.1177/070674371105600808. 21878161 10.1177/070674371105600808

[CR3] Arizona Department of Health Services (2020). Arizona Behavioral Risk Factor Surveillance System. Retrieved November 23, 2020 from https://azdhs.gov/preparedness/public-health-statistics/behavioral-risk-factor-surveillance/index.php.

[CR2] Arizona Department of Health Services (2017). Arizona Governor’s Office. Governor Ducey Declares Statewide Health Emergency in Opioid Epidemic. Retrieved April 8 from https://www.azdhs.gov/director/public-information-office/index.php#news-release-060517.

[CR4] Arizona Department of Health Services (2022). Drug Overdose Fatality Review (OFR) Team. Retrieved May 22 from https://www.azdhs.gov/prevention/womens-childrens-health/injury-prevention/index.php#ofr-team.

[CR5] Arizona Department of Health Services (2023a). OARLine. Retrieved January 3 from https://www.azdhs.gov/oarline/.

[CR6] Arizona Department of Health Services (2023b). Opioid Prevention - Opioid Overdose Deaths. Retrieved May 22 from https://www.azdhs.gov/opioid/#dashboards-overdose-deaths.

[CR7] Bhatia, D., Mikulich-Gilbertson, S. K., & Sakai, J. T. (2020). Prescription opioid misuse and risky adolescent behavior. *Pediatrics*, *145*(2). 10.1542/peds.2019-2470. 10.1542/peds.2019-2470 31907292

[CR8] Brennenstuhl, S., & Fuller-Thomson, E. (2015). The painful legacy of Childhood Violence: Migraine headaches among adult survivors of adverse childhood experiences. *Headache*, *55*(7), 973–983. 10.1111/head.12614. 26104222 10.1111/head.12614

[CR10] Cambron, C., Gringeri, C., & Vogel-Ferguson, M. B. (2014). Physical and mental health correlates of adverse childhood experiences among low-income women. *Health and Social Work*, *39*(4), 221–229. 10.1093/hsw/hlu029. 25369722 10.1093/hsw/hlu029

[CR11] Centers for Disease Control and Prevention (2021). Drug Overdose Deaths. Retrieved May 15 from https://www.cdc.gov/drugoverdose/deaths/index.html.

[CR13] Centers for Disease Control and Prevention (2022b). Understanding the Opioid Overdose Epidemic. Retrieved April 3 from https://www.cdc.gov/opioids/basics/epidemic.html.

[CR14] Centers for Disease Control and Prevention (2021). Behavioral Risk Factor Surveillance System 2020 Summary Data Quality Report. Retrieved June 20 from https://www.cdc.gov/brfss/annual_data/2020/pdf/2020-sdqr-508.pdf.

[CR15] Centers for Disease Control and Prevention (2014). About BRFSS. Retrieved December 7 from https://www.cdc.gov/brfss/about/index.htm.

[CR12] Centers for Disease Control and Prevention (2022a). Provisional Drug Overdose Death Counts. National Center for Health Statistics (US),. Retrieved May 16 from https://www.cdc.gov/nchs/nvss/vsrr/drug-overdose-data.htm.

[CR16] Christopher, P. P., Stewart, C., Manning, W., Anderson, B. J., Woodruff, A., Monteiro, & Stein, J., M. D (2022). Risk behaviors among persons civilly committed for opioid use. *Journal of Substance Abuse Treatment*, *132*(108493). 10.1016/j.jsat.2021.108493. 10.1016/j.jsat.2021.108493 PMC8627518 34098213

[CR17] Clayton, H. B., Bohm, M. K., Lowry, R., Ashley, C., & Ethier, K. A. (2019). Prescription opioid misuse Associated with Risk behaviors among adolescents. *American Journal of Preventive Medicine*, *57*(4), 533–539. 10.1016/j.amepre.2019.05.017. 31443955 10.1016/j.amepre.2019.05.017

[CR18] Colman, I., Kingsbury, M., Garad, Y., Zeng, Y., Naicker, K., Patten, S., Jones, P. B., Wild, T. C., & Thompson, A. H. (2016). Consistency in adult reporting of adverse childhood experiences. *Psychological Medicine*, *46*(3), 543–549. 10.1017/S0033291715002032. 26511669 10.1017/S0033291715002032

[CR19] Cruden, G., & Karmali, R. (2021). Opioid misuse as a coping behavior for unmet mental health needs among U.S. adults. *Drug and Alcohol Dependence*, *225*, 108805. 10.1016/j.drugalcdep.2021.108805. 34174774 10.1016/j.drugalcdep.2021.108805

[CR21] D’Amico, E. J., Davis, J. P., Tucker, J. S., Seelam, R., & Stein, B. D. (2021). Opioid misuse during late adolescence and its effects on risk behaviors, social functioning, health, and emerging adult roles. *Addictive Behaviors*, *113*(106696). 10.1016/j.addbeh.2020.106696. 10.1016/j.addbeh.2020.106696 PMC8183610 33264695

[CR20] Davis, M. A., Lin, L. A., Liu, H., & Sites, B. D. (2017). Prescription opioid use among adults with Mental Health Disorders in the United States. *Journal of the American Board of Family Medicine: Jabfm*, *30*(4), 407–417. 10.3122/jabfm.2017.04.170112. 28720623 10.3122/jabfm.2017.04.170112

[CR22] Deol, E., Siddiqui, Z., Paracha, A., Mujovic, H., Abro, Z., Chang, S. R., Abid, A., Tyler, J. H., Rehman, H., Bera, S., Khan, Z., Vasireddy, S., Chalichama, J., Fidahussain, A., Paracha, M., Ahmed, Z., Alyasiry, N., Engelhardt, M., Kadariya, B., Odeh, A., Pitchiah, M., Rao, N., Rueve, J., Cepele, I., & Meyer, D. (2023). Exploring the link between ACEs and opioid use: A systematic review. *Journal of Opioid Management*, *19*(4), 343–364. 10.5055/jom.2023.0791. 37644792 10.5055/jom.2023.0791

[CR23] Ducat, L., Philipson, L. H., & Anderson, B. J. (2014). The mental health comorbidities of diabetes. *Journal of the American Medical Association*, *312*(7), 691–692. 10.1001/jama.2014.8040. 25010529 10.1001/jama.2014.8040 PMC4439400

[CR24] Grant, B. F., Stinson, F. S., Dawson, D. A., Chou, S. P., Dufour, M. C., Compton, W., Pickering, R. P., & Kaplan, K. (2004). Prevalence and co-occurrence of substance use disorders and independent mood and anxiety disorders: Results from the national epidemiologic survey on Alcohol and related conditions. *Archives of General Psychiatry*, *61*(8), 807–816. 10.1001/archpsyc.61.8.807. 15289279 10.1001/archpsyc.61.8.807

[CR25] Guarino, H., Mateu-Gelabert, P., Quinn, K., Sirikantraporn, S., Ruggles, K. V., Syckes, C., Goodbody, E., Jessell, L., & Friedman, S. R. (2021). Adverse childhood experiences predict early initiation of opioid Use behaviors. *Front Sociol*, *6*, 620395. 10.3389/fsoc.2021.620395. 34055961 10.3389/fsoc.2021.620395 PMC8158934

[CR26] Khalili, P., Nadimi, A. E., Baradaran, H. R., Janani, L., Rahimi-Movaghar, A., Rajabi, Z., Rahmani, A., Hojati, Z., Khalagi, K., & Motevalian, S. A. (2021). Validity of self-reported substance use: Research setting versus primary health care setting. *Substance Abuse Treatment, Prevention, and Policy*, *16*(1), 66. 10.1186/s13011-021-00398-3. 34521420 10.1186/s13011-021-00398-3 PMC8439053

[CR27] Khodarahimi, S., & Fathi, R. (2016). Mental Health, coping styles, and risk-taking behaviors in young adults. *Journal of Forensic Psychology Practice*, *16*(4), 287–303. 10.1080/15228932.2016.1196101.

[CR28] Levy, S. (2019). Youth and the opioid epidemic. *Pediatrics*, *143*(2). 10.1542/peds.2018-2752. 10.1542/peds.2018-2752 30602544

[CR29] Luo, F., Li, M., & Florence, C. (2021). State-Level Economic Costs of Opioid Use Disorder and Fatal Opioid Overdose - United States, 2017. *Mmwr. Morbidity And Mortality Weekly Report*, 70(15), 541–546. 10.15585/mmwr.mm7015a1. 10.15585/mmwr.mm7015a1 PMC8344997 33857070

[CR30] Mackesy-Amiti, M. E., Donenberg, G. R., & Ouellet, L. J. (2015). Prescription opioid misuse and mental health among young injection drug users. *American Journal of Drug and Alcohol Abuse*, *41*(1), 100–106. 10.3109/00952990.2014.940424. 25105884 10.3109/00952990.2014.940424 PMC4262584

[CR31] Matjasko, J. L., Chovnick, G., Bradford, J., Treves-Kagan, S., Usher, K., Vaughn, E., & Ingoldsby, E. (2022). Strengthening communities: A qualitative Assessment of opportunities for the Prevention of adverse childhood experiences in the wake of the Opioid Crisis. *J Child Fam Stud*, *31*(4), 1145–1157. 10.1007/s10826-021-02202-z. 35002194 10.1007/s10826-021-02202-z PMC8722648

[CR32] McLaughlin, K. A., Weissman, D., & Bitrán, D. (2019). Childhood adversity and neural development: A systematic review. *Annu Rev Dev Psychol Dec*, *1*, 277–312. 10.1146/annurev-devpsych-121318-084950. 10.1146/annurev-devpsych-121318-084950 PMC7243625 32455344

[CR33] McLellan, A. T., & Woodworth, A. M. (2014). The affordable care act and treatment for substance use disorders: Implications of ending segregated behavioral healthcare. *Journal of Substance Abuse Treatment*, *46*(5), 541–545. 10.1016/j.jsat.2014.02.001. 24679908 10.1016/j.jsat.2014.02.001

[CR34] Merrick, M. T., Ford, D. C., Haegerich, T. M., & Simon, T. (2020). Adverse childhood experiences increase risk for prescription opioid misuse. *The Journal of Primary Prevention*, *41*(2), 139–152. 10.1007/s10935-020-00578-0. 31989435 10.1007/s10935-020-00578-0 PMC10976456

[CR35] Mersky, J. P., Topitzes, J., & Reynolds, A. J. (2013). Impacts of adverse childhood experiences on health, mental health, and substance use in early adulthood: A cohort study of an urban, minority sample in the U.S. *J Child Abuse & Neglect*, *37*(11), 917–929. 10.1016/j.chiabu.2013.07.011. 10.1016/j.chiabu.2013.07.011 PMC4090696 23978575

[CR36] Monnat, S. M., & Chandler, R. F. (2015). Long Term Physical Health consequences of adverse childhood experiences. *Sociol Q*, *56*(4), 723–752. 10.1111/tsq.12107. 26500379 10.1111/tsq.12107 PMC4617302

[CR37] Moriarty, D. G., Zack, M. M., & Kobau, R. (2003). The Centers for Disease Control and Prevention’s Healthy days measures- Population tracking of perceived physical and mental health over time. *Health and Quality of Life Outcomes*, *1*(37). 10.1186/1477-7525-1-37. 10.1186/1477-7525-1-37 PMC201011 14498988

[CR38] National Center for Injury Prevention and Control, Division of Violence Prevention (2023). Behavioral Risk Factor Surveillance System ACE Data. Centers for Disease Control. https://www.cdc.gov/violenceprevention/aces/ace-brfss.html.

[CR39] National Institute on Drug Abuse (2020). Criminal Justice Drug Facts. Retrieved June 20 from https://nida.nih.gov/download/23025/criminal-justice-drugfacts.pdf?v=25dde14276b2fa252318f2c573407966.

[CR40] Quello, S. B., Brady, K. T., & Sonne, S. C. (2005). Mood disorders and substance use disorder: A complex comorbidity. *Science & Practice Perspectives / a Publication of the National Institute on Drug Abuse, National Institutes of Health*, *3*(1), 13–21. 10.1151/spp053113. 10.1151/spp053113 PMC2851027 18552741

[CR41] Quinn, K., Boone, L., Scheidell, J. D., Mateu-Gelabert, P., McGorray, S. P., Beharie, N., Cottler, L. B., & Khan, M. R. (2016). The relationships of childhood trauma and adulthood prescription pain reliever misuse and injection drug use. *Drug and Alcohol Dependence*, *169*, 190–198. 10.1016/j.drugalcdep.2016.09.021. 27816251 10.1016/j.drugalcdep.2016.09.021 PMC5728665

[CR42] Reuben, A., Moffitt, T. E., Caspi, A., Belsky, D. W., Harrington, H., Schroeder, F., Hogan, S., Ramrakha, S., Poulton, R., & Danese, A. (2016). Lest we forget: Comparing retrospective and prospective assessments of adverse childhood experiences in the prediction of adult health. *Journal of Child Psychology and Psychiatry*, *57*(10), 1103–1112. 10.1111/jcpp.12621. 27647050 10.1111/jcpp.12621 PMC5234278

[CR43] Reynolds, E. K., Schreiber, W. M., Geisel, K., MacPherson, L., Ernst, M., & Lejuez, C. W. (2013). Influence of social stress on risk-taking behavior in adolescents. *Journal of Anxiety Disorders*, *27*(3), 272–277. 10.1016/j.janxdis.2013.02.010. 23602940 10.1016/j.janxdis.2013.02.010 PMC3693744

[CR44] Rosenbaum, R., & Kandel, D. B. (1990). Early Onset of adolescent sexual behavior and drug involvement. *Journal of Marriage and Family*, *52*(3), 783–798. 10.2307/352942.

[CR45] Ruengvirayudh, P., & Brooks, G. P. (2016). Comparing stepwise regression models to the best-subsets models, or, the art of stepwise. *General Linear Model Journal*, *42*(1), 1–14.

[CR46] Sanchez-Roige, S., Fontanillas, P., Jennings, M. V., Bianchi, S. B., Huang, Y., Hatoum, A. S., Sealock, J., Davis, L. K., Elson, S. L., andMe Research, T., & Palmer, A. A. (2021). Genome-wide association study of problematic opioid prescription use in 132,113 23andMe research participants of European ancestry. *Molecular Psychiatry*, *26*(11), 6209–6217. 10.1038/s41380-021-01335-3. 34728798 10.1038/s41380-021-01335-3 PMC8562028

[CR47] Schuckit, M. A. (2016). Treatment of Opioid-Use disorders. *New England Journal of Medicine*, *375*(4), 357–368. 10.1056/NEJMra1604339. 27464203 10.1056/NEJMra1604339

[CR48] Schwartz, A., Arsandaux, J., Montagni, I., Meschke, L. L., Galera, C., & Tzourio, C. (2023). Adverse childhood experiences and substance use among university students: A systematic review. *Journal of Substance Use*, 1–11. 10.1080/14659891.2022.2114389.

[CR49] Seal, K. H., Shi, Y., Cohen, G., Cohen, B. E., Maguen, S., Krebs, E. E., & Neylan, T. C. (2012). Association of mental health disorders with prescription opioids and high-risk opioid use in US veterans of Iraq and Afghanistan. *Journal of the American Medical Association*, *307*(9), 940–947. 10.1001/jama.2012.234. 22396516 10.1001/jama.2012.234

[CR50] Smith, G. (2018). Step away from stepwise. *J Big Data*, *5*(32). 10.1186/s40537-018-0143-6.

[CR51] Sofuoglu, M., DeVito, E. E., & Carroll, K. M. (2018). Pharmacological and behavioral treatment of opioid Use Disorder. *Psychiatric Research & Clinical Practice*, *1*(1), 4–15.

[CR52] Stein, M. D., Conti, M. T., Kenney, S., Anderson, B. J., Flori, J. N., Risi, M. M., & Bailey, G. L. (2017). Adverse childhood experience effects on opioid use initiation, injection drug use, and overdose among persons with opioid use disorder. *Drug and Alcohol Dependence*, *179*, 325–329. 10.1016/j.drugalcdep.2017.07.007. 28841495 10.1016/j.drugalcdep.2017.07.007 PMC5599365

[CR53] Stotts, A. L., Dodrill, C. L., & Kosten, T. R. (2009). Opioid dependence treatment: Options in pharmacotherapy. *Expert Opinion on Pharmacotherapy*, *10*(11), 1727–1740. 10.1517/14656560903037168. 19538000 10.1517/14656560903037168 PMC2874458

[CR54] Substance Abuse and Mental Health Services (2005). In Medication-Assisted Treatment for Opioid Addiction in Opioid Treatment Programs. https://www.ncbi.nlm.nih.gov/pubmed/22514849. 22514849

[CR55] Sullivan, M. D., Edlund, M. J., Zhang, L., Unutzer, J., & Wells, K. B. (2006). Association between mental health disorders, problem drug use, and regular prescription opioid use. *Archives of Internal Medicine*, *166*(19), 2087–2093. 10.1001/archinte.166.19.2087. 17060538 10.1001/archinte.166.19.2087

[CR56] Swedo, E. A., Sumner, S. A., de Fijter, S., Werhan, L., Norris, K., Beauregard, J. L., Montgomery, M. P., Rose, E. B., Hillis, S. D., & Massetti, G. M. (2020). Adolescent opioid misuse attributable to adverse childhood experiences. *Journal of Pediatrics*, *224*, 102–109e103. 10.1016/j.jpeds.2020.05.001. 32437756 10.1016/j.jpeds.2020.05.001 PMC8253221

[CR57] Trenz, R. C., Scherer, M., Harrell, P., Zur, J., Sinha, A., & Latimer, W. (2012). Early onset of drug and polysubstance use as predictors of injection drug use among adult drug users. *Addictive Behaviors*, *37*(4), 367–372. 10.1016/j.addbeh.2011.11.011. 22172686 10.1016/j.addbeh.2011.11.011 PMC3288417

[CR58] Truth Initiative (2023). Who we are. Retrieved December 8, 2023 from https://truthinitiative.org/what-we-do/youth-smoking-prevention-education.

[CR59] VanderEnde, K., Chiang, L., Mercy, J., Shawa, M., Hamela, J., Maksud, N., Gupta, S., Wadonda-Kabondo, N., Saul, J., Gleckel, J., Kress, H., & Hillis, S. (2018). Adverse childhood experiences and HIV sexual risk-taking behaviors among young adults in Malawi. *J Interpers Violence*, *33*(11), 1710–1730. 10.1177/0886260517752153. 29739289 10.1177/0886260517752153 PMC6158791

[CR60] Webster, L. R. (2017). Risk factors for opioid-use disorder and overdose. *Anesthesia and Analgesia*, *125*(5), 1741–1748. 10.1213/ANE.0000000000002496. 29049118 10.1213/ANE.0000000000002496

[CR61] Wu, L. T., Zhu, H., & Ghitza, U. E. (2018). Multicomorbidity of chronic diseases and substance use disorders and their association with hospitalization: Results from electronic health records data. *Drug and Alcohol Dependence*, *192*, 316–323. 10.1016/j.drugalcdep.2018.08.013. 30312893 10.1016/j.drugalcdep.2018.08.013 PMC6358206

