# Supplementary material for: Association Between Self-Reported Opioid Use and Behavioral/Social Health Characteristics—Arizona, 2020
Source: Int J Ment Health Addict. 2024 Mar 18;23(4):3030–45. doi: 10.1007/s11469-024-01276-2 (PMC12423179; doi:10.1007/s11469-024-01276-2)
Supplement: Supplementary file 1 — Supplementary Material 1 [file 11469_2024_1276_MOESM1_ESM.docx]

**Supplemental Table 1.** Unadjusted Odds Ratios (OR) and 95% Confidence Intervals (95% CI) of Three Self-Reported Opioid Misuse Measures, BRFSS, Arizona, 2020

|  | **Model 1: Misuse of Prescription Pain Medication**  N=160, N_weighted_=115,277 | | **Model 2: Injection of non-prescription drugs**  N=29, N_weighted_=23,993 | | **Model 3: Dependent on prescription pain medication**  N=51, N_weighted_=26,540 | |
| --- | --- | --- | --- | --- | --- | --- |
|  | **OR** | **95% CI** | **OR** | **95% CI** | **OR** | **95% CI** |
| **Number of ACEs** | | | | | | |
| 0 | 1 | Ref. | 1 | Ref. | 1 | Ref. |
| 1 | 2.0 | (1.0, 3.9) | 2.6 | (0.6, 10.8) | 0.9 | (0.1, 8.5) |
| 2-3 | 5.5 | (3.1, 9.7) | 5.0 | (1.2, 20.4) | 2.7 | (0.6, 11.6) |
| 4+ | 9.9 | (5.7, 17.0) | 11.6 | (3.2, 41.4) | 9.7 | (2.4, 38.7) |
| **Mental health^a^** | | | | | | |
| Not poor | 1 | Ref. | 1 | Ref. | 1 | Ref. |
| Poor | 2.7 | (1.6, 4.7) | 10.5 | (3.8, 29.0) | 6.6 | (2.5, 17.6) |
| **Number of Risk-Taking Behaviors^b^** | | | | | | |
| 0 | 1 | Ref. | 1 | Ref. | 1 | Ref. |
| 1 | 1.5 | (0.9, 2.5) | 10.6 | (1.9, 61.3) | 1.2 | (0.4, 3.9) |
| 2+ | 5.4 | (3.4, 8.8) | 18.2 | (3.3, 99.7) | 2.5 | (0.8, 7.5) |

Abbreviations: OR- Odds Ratio, CI- Confidence Interval, ACE- Adverse Childhood Event, RTB- Risk-Taking Behavior

Model 1: Logistic regression of ACEs, Mental Health, or Risk-Taking Behaviors and prescription pain medication misuse

Model 2: Logistic regression of ACEs, Mental Health, or Risk-Taking Behaviors and injection of non-prescription drugs

Model 3: Logistic regression of ACEs, Mental Health, or Risk-Taking Behaviors and dependency on prescription pain medication

Definitions: ^a^Poor Mental Health- Reported 14+ days mental health was not good in the last 30 days or Reported ever being told you had a depressive disorder (including depression, major depression, dysthymia, or minor depression); ^b^Risk-Taking Behavior- Current or former smoker, does not always wear a seatbelt, history of drinking and driving, binge drinking in the past 30 days, or marijuana use in the past year; Misuse of Prescription Pain Medication: Survey participants answered “Yes” to “In the past year, did you use a prescription pain medication that was not prescribed specifically for you by a doctor, dentist, nurse practitioner, or healthcare providers?”; Injection of non-prescription drugs: Survey participants answered “Yes” to “In the past 12 months, did you shoot up or inject any drugs other than those prescribed for you?”; Dependent on prescription pain medication: Survey participants answered “Yes” to “In the past year have you felt dependent on prescription pain medication or experienced trouble getting off of the medication when you no longer needed it for medical reasons?”
